# A Qualitative Study Exploring Rehabilitant and Informal Caregiver Perspectives of a Challenging Rehabilitation Environment for Geriatric Rehabilitation

**DOI:** 10.1177/23743735231151532

**Published:** 2023-01-17

**Authors:** Lian M J Tijsen, Els W C Derksen, Wilco P Achterberg, Bianca I Buijck

**Affiliations:** 1 Department of Public Health and Primary Care, Leiden University Medical Center, Leiden, The Netherlands; 2 Oktober, Bladel, The Netherlands; 3 De Zorgboog, Bakel, The Netherlands; 4 Department of Primary and Community Care, Radboud University Medical Center, Nijmegen, The Netherlands

**Keywords:** challenging rehabilitation environment, geriatric rehabilitation, informal caregiver, rehabilitant

## Abstract

There is a trend toward formalization of the rehabilitation process for older rehabilitants in a Challenging Rehabilitation Environment (CRE). This concept involves the comprehensive organization of care, support, and environment in rehabilitation wards. So far, literature about the principles of CRE is scarce. This study aims to explore the opinions of rehabilitants and informal caregivers regarding CRE, through a qualitative study between 2019 and 2020. Three telephone interviews were conducted with informal caregivers, and also 3 focus groups with 15 rehabilitants and 3 informal caregivers, all with recent experience in rehabilitation. Nine themes emerged regarding the rehabilitation process: (1) rehabilitant (attention for resilience, motivation, cognitive and emotional aspects), (2) rehabilitant centered (goal setting, physical and cognitive functioning and coping), (3) informal caregivers (involving and attention for resilience and relation), (4) communication (aligning the rehabilitation process), (5) exercise (increasing intensity by using task-oriented exercise, patient-regulated exercise, and group training), (6) peer support (learning experiences and recognition), (7) daily schedule (influence on the planning and activities outside therapy), (8) nutrition (energy for rehabilitation), and (9) eHealth (makes rehabilitation more challenging and fun). Regarding organizational processes, 4 themes were identified: (1) environmental aspects (single bedrooms, shared room for activities and therapy options on the ward), (2) staff aspects (small team with an emphatic supportive and motivating attitude), (3) organizational aspects (organized in an efficient way), and (4) return home (the discharge process should be well prepared for instance with home visits). Organizing excellent rehabilitation care requires a thorough understanding of the concept of CRE, as it is a complex and comprehensive concept that concerns the whole rehabilitation process. Its effectiveness and efficiency should be researched in prospective studies.

## Introduction

The demand for Geriatric Rehabilitation (GR) in Europe increases with the aging of the population (1). After hospitalization, 11% of those aged ≥ 75 years are referred to a postacute geriatric rehabilitation unit (2). Common reasons for hospitalization of older persons are cardiac events, infections, fall-related injuries, stroke, cancer, or medical/surgical interventions (3). In 2019, 53 320 rehabilitants in the Netherlands were referred for GR (4).

GR is defined as “a multidimensional approach of diagnostic and therapeutic interventions, the purpose of which is to optimize functional capacity, promote activity and preserve functional reserve and social participation in older people with disabling impairments (5).”

Persons who are rehabilitating are trying to adapt and self-manage their current condition. In line with the ideas on positive health of Huber et al, we have chosen not to label persons during their rehabilitation as patients, but to use the term “rehabilitants” when referring to them (6).

A Challenging Rehabilitation Environment (CRE) is a relatively new concept which involves the comprehensive organization of care and support by the rehabilitation team, as well as the environment in which the rehabilitation takes place (7,8). Knowledge about this concept may improve the rehabilitation outcomes of rehabilitants, for example, in terms of functional recovery, length of stay, (health-related) quality of life, and lower caregiver burden (8).

In a review, 7 main components for modeling CRE were identified: (1) therapy time, (2) group training, (3) patient-regulated exercise, (4) family participation, (5) task-oriented training, (6) enriched environment, and (7) team dynamics (8).

Because there is no official definition of CRE, there are considerable differences between wards in the interpretation and execution of CRE. Therefore, it is not certain that the beforementioned components cover all aspects of CRE. In addition, it is unknown which components rehabilitants and their informal caregivers consider relevant for a CRE. The aim of rehabilitation is to optimize the level of functioning of a rehabilitant. The availability of an informal caregiver during and after the rehabilitation is beneficial for the rehabilitation outcomes (8). Therefore, it is essential to know their vision of an optimal rehabilitation environment.

The aim of the CREATE study (Challenging REhAbiliTation Environment) is to substantiate the added value of the concept of CRE (9). In this study, we, therefore, explore the perspectives of rehabilitants and their informal caregivers regarding components that are relevant for modeling and understanding the principles of a CRE. By integrating the evidence found in the literature and the perspectives of rehabilitants, informal caregivers, and professionals, an evidence-based conceptualization of CRE can be developed.

## Methods

### Study Design

A qualitative study, consisting of focus groups and telephone interviews, was performed between September 2019 and October 2020. The aim of this research was to explore the perspectives of (older) rehabilitants and informal caregivers on the concept of CRE. Qualitative research attains to gain a better understanding of a phenomenon through the experiences of those involved (10).

We adhered to the consolidated criteria for reporting qualitative research (COREQ) for improving the quality of reporting qualitative research (see Supplemental Table) (11).

### Recruitment of Participants

Organizations affiliated with the 6 scientific networks (living labs) for elderly care in the Netherlands demonstrate an interest in scientific research. Therefore, these organizations were approached by email with information about this study (12). They were requested to inform rehabilitants and their informal caregivers about the study, and to ask them to individually participate. In addition, members of a patient association for patients with acquired brain injury “Hersenletsel,” were asked to participate (13). We aimed at a mix of participants with different diagnoses and ages.

Rehabilitants and informal caregivers were included if they were currently participating or had recently participated in a (geriatric) rehabilitation process. Rehabilitants and informal caregivers were excluded if they were diagnosed with dementia, were (legally) unable to give informed consent, or were not proficient in the Dutch language.

If rehabilitants and/or their caregivers were interested in the study, they received an information leaflet. If they remained interested or had additional questions, they were put in contact with the primary researcher (LT). Questions were answered, and the informed consent form was signed by the participant and LT. If an informal caregiver participated without the rehabilitant, the rehabilitant was asked for permission to interview the informal caregiver. An appointment was then made for a focus group or telephone interview.

We aimed for data saturation, and after each focus group or interview, the authors discussed whether any new topics had emerged. Inclusion stopped when no new topics emerged.

### Data Collection

In preparation for the data collection, a topic list was established based on the above-mentioned review (Appendix 1) (8). This topic list was piloted with a group of researchers. The content of the list was determined in an iterative process and adapted based on the pilot and previous focus groups or interviews.

The focus group interviews were chaired by BB and LT. Female nursing senior researcher in the field of rehabilitation BB has experience in qualitative research and chairing discussion groups. Physical therapist LT is a female PhD student with formal training in interview techniques and qualitative research and 10 years of experience in geriatric rehabilitation.

The focus groups were held on rehabilitation wards in various regions in the Netherlands. All focus groups were performed in meeting rooms and only the participants and researchers were present.

Due to COVID-19, we switched from focus group meetings to individual, semi-structured, telephone interviews with informal caregivers during the data collection. These interviews were conducted by LT, using the same topic list.

Each focus group and telephone interview began with a brief introduction by the researchers on the topic of the focus group, followed by an introduction of the participants. The participants were then asked to share their perspectives on CRE. The chair used open-ended questions based on the topic list and further explored the answers that were given. To increase the internal validity, participants were also asked to share their perspectives on subjects not mentioned in the topic list, but which they considered important regarding CRE. During each focus group and interview, LT made field notes.

On average the focus groups lasted 90 min and the telephone interviews 45 min. Both were audio recorded and transcribed verbatim by LT. Transcripts were not returned to participants, but at the end of every interview, the chair verified a verbal summary with the participants.

### Data Analysis

Simultaneous with the data collection, thematic analysis was performed to identify, analyze, and report patterns in the data (10,14). ATLAS.ti version 7.5 was used for coding the data in the analysis process.

LT read and re-read the data to become familiar with the data, after which initial themes were identified while reading through the transcript using an open-coding approach. These initial themes were checked by BB and ED to determine inter-rater comparisons. ED is a female nursing senior researcher with experience in qualitative research.

Differences in the coding were discussed by LT, ED, and BB until an agreement was reached. Each initial theme was described in a memo.

The identified initial themes were combined into main themes with associated subthemes. The connections and contradictions between the initial themes were described per the main theme, and connections between main themes were described.

Each main theme was assessed for data saturation, after which the research team discussed the contents of the main themes. After the agreement was reached with the research team, each main theme was meticulously described, and relevant quotes were identified and translated from Dutch into English.

## Results

### Participants

The approached rehabilitants (15) and informal caregivers (6) all agreed to participate. In September and October 2019, a total of 3 focus group interviews were conducted, and in September and October 2020, 3 telephone interviews were held with informal caregivers. Rehabilitants were between 43 and 90 years old and informal caregivers were between 49 and 77 years old. Rehabilitation diagnoses were lower limb amputation, trauma, hip fracture, chronic obstructive pulmonary disease (COPD), and acquired brain injury, including stroke. Characteristics of the participants are shown in Table 1.

**Table 1.** Characteristics of Participants.

		Rehabilitants (n = 15)	Informal caregivers (n = 6)
Age	Range (median)	43-90 (75)	49-77 (72)
Gender	Male	9	2
	Female	6	4
Diagnosis	Lower limb amputation	2	
	Trauma, excl. hip fracture	5	1
	Hip fracture	1	
	COPD	1	
	Acquired brain injury, incl. stroke	6	5
Relationship	Spouse		5
	Daughter		1

Abbreviation: COPD, chronic obstructive pulmonary disease.

### Themes

Thirteen main themes with associated subthemes emerged from the data. The main themes can be divided into 2 categories, namely themes involving the rehabilitation process and themes involving organizational processes. The subdivision of the themes in the 2 categories is described in Tables 2 and 3. The 2 categories are described in the following paragraphs.

**Table 2.** Themes Involving Rehabilitation Process.

Main theme	Brief description	Subtheme	Description
Rehabilitant	CRE is suitable for all diagnosis groups. Attention must be given to resilience, motivation, and cognitive and emotional aspects of rehabilitants.	Resilience	The resilience of rehabilitants can vary and may differ from prediagnosis. Rehabilitants would like guidance on how to deal with this, as they often tend to go beyond their limits.
		Motivation	Participants think being motivated for rehabilitation is important. Motivation differs per rehabilitant. Working on individual rehabilitation goals, the strong involvement of professionals and the use of technologies can be motivating.
		Cognitive and emotional aspects	Rehabilitants feel frustrated when dependent on professionals and informal caregivers. Especially in the case of neurological diagnoses, rehabilitants do not always feel others empathize with what they are going through. They would like more consideration for and better explanation of their problems, for themselves and also for their caregivers. These problems include, for example, altered stimulus processing, overburden, a decline of executive functions, dealing with emotions, loss of memory, and loss of initiative.
		Diagnosis	CRE is suitable for all diagnoses. Although rehabilitants appreciate having rehabilitants with similar diagnoses on the ward, they do not consider complete differentiation based on diagnosis necessary.
Rehabilitant-centered process	The rehabilitation process should be tailored to a rehabilitants situation. This includes the goal-setting process, the level of physical and cognitive functioning, and coping with the life event for which they are rehabilitating.	Goal setting	Rehabilitation goals must fit seamlessly with the rehabilitants’ situation. Although rehabilitants think that working on their individual goals is motivating, they do not always feel involved in the goal-setting process. Some rehabilitants may not be able to set individual goals at the start of their rehabilitation and most of them value the opinion of the professionals. Participants agree that some goals are better achieved at home, not during inpatient rehabilitation.
		Tailor-made rehabilitation	Rehabilitation must be tailored to the rehabilitant's abilities. This tailoring depends on several factors, which sometimes also existed before rehabilitation, for example, the level of physical and cognitive functioning. Rehabilitants with the ability to manage the intensity and planning of the rehabilitation valued this positively.
		Coping with a life event	The coping process, which can be compared to a grieving process, can influence goal setting and continues after discharge. Rehabilitants and their caregivers must regain confidence in themselves. Contact with fellow rehabilitants and guidance from professionals, for example, a psychologist, can help with coping.
Informal caregivers	Informal caregivers should be involved in the rehabilitation process with attention to their resilience and the relation between the rehabilitant and informal caregiver.	Informal caregiver participation	Informal caregiver participation can have a beneficial effect because caregivers learn what to do at home. But it can also be inhibiting if the caregiver does not trust the rehabilitant. Attention to the resilience of the caregiver is important, and they must be continuously involved in the rehabilitation process. Informal caregivers value doing extra exercises with the rehabilitant and, if needed, are part of the conversation regarding the rehabilitation process.
		Dynamic between rehabilitant and informal caregiver	The role of the caregiver during rehabilitation can depend on the relationship with the rehabilitant. A partner may have a more natural role than another informal caregiver, such as a child. Caregivers consider it quite normal to do extra things for their spouse. However, it is important that the relationship does not become a care relationship only.
Communication	Communication is important for aligning the rehabilitation process between all involved in the process.		Adequate communication is important for building a treatment relationship, providing mutually relevant information about the disease, aligning goals, communicating exercise options, preparation for the discharge process and the coping process. Participants value reading the reports and the goals in the patient files. Special attention is needed if the rehabilitant cannot oversee his rehabilitation process, for instance, due to cognitive impairment, or if the caregiver is unable to come to the ward due to circumstances like COVID-19. Repeating the information is an important aspect.
Exercise	Exercise intensity in a CRE should be as high as possible. This can be achieved by integrating task-oriented exercises, patient-regulated exercises, and group training into the daily structure.	Therapeutic activity	Although the therapeutic activity varies, most participants indicate they would like to see a higher therapy intensity, with guidance to help avoid overburdening. Participants see a role for informal caregivers and nurses to stimulate extra therapeutic activity.
		Group training	Rehabilitants who had experienced it are very positive about group training and find it motivating and helpful to learn from each other. Those without experience in group training cannot imagine how it can be tailored to a rehabilitant's individual goals and situation. They expect it to be less intensive than individual training.
		Task-oriented exercise	Rehabilitants think doing task-oriented exercises, matching their goals and level of functioning, is important. Nurses can be supportive in task-oriented exercises. Rehabilitants want to be independent and need professionals to guide them in safe independent practice training during their daily routine.
		Patient-regulated exercise	Rehabilitants do think patient-regulated exercise is important to increase the therapy intensity, but they need guidance. Exercises need to fit rehabilitant needs, and follow-up by professionals is desired. Rehabilitants have concerns regarding overburden, practicing at insufficient intensity, and safety when training individually in therapy rooms without supervision.
Peer support	Peer support is important for learning experiences, putting things in perspective, and recognition.		Most rehabilitants think peer support is important for support, putting things in perspective, and recognition. They feel that they are not alone with these problems. Rehabilitants can learn from each other and practicing together can be motivating. Rehabilitants can have a positive or negative effect on the group dynamic.
Daily schedule	Rehabilitants want to have input in the therapy planning and would value activities outside therapy moments.	Planning	Rehabilitants prefer a therapy planning in which they have input, such as in terms of intensity, therapy moments should be spread throughout the day. This planning can help them to plan visits and avoid distractions during therapy moments.
		Activities during the day	Besides the therapy moments, participants experience a limited number of activities, which does not stimulate a sense of active rehabilitation. As rehabilitants are not always able to start an activity themselves, they would value (therapeutic) activities outside therapy moments. They also have a need to continue social activities as usual.
Nutrition	Nutrition gives energy for the rehabilitation process, and mealtimes can stimulate contact between rehabilitants.		Good and tasty nutrition is one of the first aspects participants mention as being important during rehabilitation. Nutrition gives energy to the rehabilitation process. If compatible with their goal, rehabilitants think that mealtimes can play a role in task-oriented training. A pleasant mealtime with interaction stimulates contact between rehabilitants and makes rehabilitants feel less lonely.
eHealth	eHealth can make rehabilitation more fun and challenging.		Participants’ opinions on eHealth differ regarding their use of it. They may not be using it yet but expect that it will be important for future generations. The use of eHealth must suit the person. eHealth can make the rehabilitation process more fun and can stimulate exercise. It offers communication options in case of aphasia and when used properly, technologies can increase safety, for example in the home situation.

Abbreviation: CRE, Challenging Rehabilitation Environment.

**Table 3.** Themes Involving Organizational Processes.

Main theme	Brief description	Sub-theme	Description
Environmental aspects	A rehabilitation ward should have single bedrooms, a shared room for activities, the possibility to go outside, and therapy options on the ward.	Location of rehabilitation	Rehabilitation should take place close to their own home, in a central location that is easy for people to visit, and also offers other activities so rehabilitants can go shopping. They would rather not rehabilitate in a nursing home with permanent residents, which confronts them with suffering.
		Building aspects	Rehabilitation should take place in small wards with a shared room for activities and mealtimes, and the possibility to go outside. Most participants would like to see therapy options on the ward. The therapy room must have sufficient space, good climate control, and must be easy to adjust based on their needs. Chairs should be provided in long corridors. Rehabilitants with acquired brain injury want attention for low-stimulus areas to avoid overstimulation.
		Bedrooms	All participants want a single bedroom, with a private bathroom. They see benefits to having privacy in the areas of exercise options, night rest, privacy, hygiene, and for coping with everything that is happening.
		Tools	On a rehabilitation ward, there must be sufficient and practical aids, like exercise materials, walking aids, lifting aids, and eating and drinking aids. Bicycles, a walking circuit, relevant games, a tablet with aphasia programs, and walking rails are named as examples of desired exercise materials.
Staff aspects	The rehabilitation facility should have small teams with good mutual communication. All team members should have an empathic way of supporting and motivating rehabilitants.	Staff	Staff must guide rehabilitants in their rehabilitation process in an empathic way, both in terms of emotional support and in exercising. They need knowledge, provide information, have an active attitude, communicate well and stimulate or, if necessary, slow rehabilitants down. A good relationship with staff can have a stimulating effect on the rehabilitant.
		Team	Participants prefer small teams with good mutual communication. Team members need to be aware of rehabilitation (needs) and strive for sufficient collaboration with other team members. Mentioned team members are physical therapist, occupational therapist, psychologist, nurse, physician, speech therapist, dietician, social worker and recreational therapist. Physical therapy is named as the most important discipline; some participants feel resistance to the psychologist. Participants do see a therapeutic role for nurses in practicing activities of daily living, providing support, and stimulating additional exercises.
Organizational aspects	Rehabilitation should be organized in an efficient way to optimize the results.		Participants feel that financial cutbacks lead to a high workload for staff and a shorter length of stay, which has adverse consequences for their rehabilitation. They do feel it is important to organize the rehabilitation process in an efficient way, to optimize the results of the rehabilitation.
Return home	The discharge process must be well-prepared and supervised. Home visits allow rehabilitants to practice meaningful tasks in their own environment in preparation for their discharge.	Home visit	Home visits during the rehabilitation process are effective to see whether it is possible to function at home and which home adjustments are needed. However, confrontation with (un)expected disabilities can make a home visit emotionally challenging.
		Discharge process	Although some participants think the rehabilitation process is going too fast, most want to keep their time on the ward as short as possible. Good and timely communication about this process is important.
		Collaboration with external care professionals	It is important for transfers between the different settings in a rehabilitation process to take place in a good and timely manner. Participants who have experienced a gap in this process feel they have deteriorated because of it. Participants need advice from (community) therapists with sufficient experience to continue the rehabilitation after discharge.
		Outpatient rehabilitation	Rehabilitants are not sure if their rehabilitation would be faster at home or on a rehabilitation ward. They are all aware that the rehabilitation process continues after discharge. Therapy intensity at home can depend on the funding of health care insurers.

#### Category 1: Themes Involving Rehabilitation Process

This category consists of 9 main themes, namely (1) rehabilitant, (2) rehabilitant centered, (3) informal caregivers, (4) communication, (5) exercise, (6) peer support, (7) daily schedule, (8) nutrition, and (9) eHealth.

##### Theme 1.1: Rehabilitant

Rehabilitants must learn to deal with changing resilience. Participants agree that some rehabilitants have to be motivated for rehabilitation, while others need slowing down. As one rehabilitant with acquired brain injury said,

Because of course there are two extremes. For some things you have to be pulled out of bed, because you don't want to get out of bed, but you have to anyway. And other people start sprinting very quickly, while in fact well … maybe you shouldn't.

Participants experience feelings of dependency and changes in cognitive and emotional aspects. According to a rehabilitant with a stroke, it is important to provide explanations about these changes to both the rehabilitant and his informal caregivers,

But even with people who are very close, who really know what is going on etc. Well, I still run into things. Then I think yes … even they don't quite get this.

Rehabilitants value having peers with similar diagnoses in a rehabilitation ward. But, as one rehabilitant with acquired brain injury said, complete differentiation on diagnoses is not necessary,

I do agree with you that you really want to be in a like-minded group. But I want to decide for myself where to sit.

##### Theme 1.2: Rehabilitant-Centered Process

The rehabilitation process must be tailored to the individual goals, abilities, and situation. Working on individual goals can be motivating, but not all rehabilitants are able to set their goals at the beginning of the process, as one rehabilitant with acquired brain injury said,

But in the beginning, it is so important that you have some kind of clarity, of where you want to go. And that those goals are adjusted every time. In the beginning well … when you start something, you have no idea what that is.

This can be influenced by a grieving process, through which a rehabilitant and his informal caregivers need to cope with this life event. In the words of a rehabilitant with a traumatic arm fracture,

I had that last Saturday. I broke down  … And they stayed with me for a bit. Yes. Then you have to get over it, put it behind you.

##### Theme 1.3: Informal Caregivers

Involvement of the informal caregiver in the rehabilitation process can have a positive effect. Participants see benefits in terms of increasing understanding of the situation of the rehabilitant, creating confidence, and providing additional exercise moments. As a rehabilitant with a lower limb amputation said,

And then, for example, I went home last weekend … And then she does things that annoy me, that I don't think are necessary. But she's so scared because she doesn't know what I am able do. But if they've already helped here a few times, then they gain confidence in the person.

And as an informal caregiver said about his wish to exercise together with his spouse,

Of course, therapy doesn't go on all day. So, if you can overcome that yourself by doing other exercises. That sounds like a good idea, yes.

The extent to which informal caregivers can provide support is determined by their resilience, but also by their relationship with the rehabilitant. As an informal caregiver explained, it is often quite normal for a spouse to be involved in the rehabilitation process,

Well, look, you hear the word “informal carer” a lot. But I do not consider myself a carer … For my wife, who is going through a difficult time. You just do a bit more, no big deal.

##### Theme 1.4: Communication

Adequate communication is important, as a rehabilitant with acquired brain injury mentioned,

But I think the most important thing in the whole rehabilitation process is to communicate. Look at the person in front of you. Really look. Because that's … And I think 80% of the time it's OK. But 20% of the time it hurts so much.

As an informal caregiver explained, special attention is needed for communication with the informal caregivers, especially if the rehabilitant cannot oversee his rehabilitation process,

Yes, I just wanted an impression of how she was doing. She wasn’t able to provide that.

As a rehabilitant with acquired brain injury indicated, it is also important to provide information about the disease,

I thought it was very important that I got a lot of explanations about what's wrong. That it was explained. So I could understand myself.

##### Theme 1.5: Exercise

Most participants would like to see a higher intensity of therapeutic activities. One way to achieve this is group training. Participants with experience in group training are very positive about it, like this rehabilitant with a traumatic fracture,

I think the advantages are that you see that you are not the only one stumbling around and tired after cycling for a little while. And that does tell you, you think well he can do it, so I can do it too. That is encouraging.

But rehabilitants without experience in group training cannot see how it can be tailored to a rehabilitant's needs, like this rehabilitant with a lower limb amputation,

Not if it is not tailored specifically to the patient, then group therapy is not … If it's all the same patients …

Participants see an important role for nurses in stimulating and supporting rehabilitants by practicing meaningful tasks outside therapy moments. As one rehabilitant with traumatic fracture said, therapy moments can be created in all daily activities,

But going alone is always therapy. So also sitting down in the chair and everything. At some point all of it is therapy.

Rehabilitants think it is important to do patient-regulated exercises, but they want advice concerning the safety of the exercises and the prevention of overburden. For example this rehabilitant with a traumatic fracture, who thinks rehabilitants can be afraid to practice alone in the therapy room,

I would love to, but then I would want someone there to see what you are going to do. I wouldn't dare to do it on my own, just …

##### Theme 1.6: Peer Support

Peer support is important for support, motivation, learning, putting things in perspective, and recognition. As a rehabilitant with acquired brain injury said,

And that's why it's so nice to be with people who have the same problem and you don't have to explain anything, they just understand. That always feels so relaxed.

##### Theme 1.7: Daily Schedule

Participants prefer to have input in the planning of their therapy moments and have these moments well spread in intensity throughout the day. In addition to therapy, they would appreciate the possibility to do activities, as an informal caregiver explained,

You could see it in several people. That, in fact, outside therapy there was a sense of aimlessness. It wasn't the active feeling of “I'm doing rehab now.”

##### Theme 1.8: Nutrition

Attention to good and tasty nutrition is important in a rehabilitation process, as one rehabilitant with a lower limb amputation explained,

Eating well is also therapy. You need the energy to be able to do your therapy. I need my breakfast and my food for the diabetes. But I also need to do therapy.

Mealtimes can play a role in task-oriented training but are also important to stimulate contact between rehabilitants and create a good ambiance. As a rehabilitant with acquired brain injury mentioned,

But I think that for some people, because of course their rehabilitation takes a long time and they are alone … Then it's nice when you don't feel that you're eating alone on top of all that.

##### Theme 1.9: eHealth

eHealth is defined as “the use of digital information and communication to support and/or improve health and health care (15).” Participants do think eHealth can make the rehabilitation process more fun, stimulate doing exercises, and provide safety. But they do not think eHealth is always a necessary add-on to the rehabilitation process, as this informal caregiver summarized,

Depending on what those technical things are for, of course. If the technical things contribute more to the recovery of the patient than the non-technical things, then I am all for the technical things. But if it doesn't add much value, then I think well …

#### Category 2: Themes Involving Organizational Processes

This category consists of 4 main themes, namely (1) environmental aspects, (2) staff aspects, (3) organizational aspects, and (4) return home.

##### Theme 2.1: Environmental Aspects

A rehabilitation center should be close to the residence of a rehabilitant. The wards should be small, with a shared living room and the possibility to go outside. The wards should provide practice options for the rehabilitants, such as aids required for rehabilitation, but also sufficient space to practice. As an informal caregiver said, it would be helpful if the therapy rooms are integrated into the ward,

I imagine a rehabilitation department, which is fully integrated. Everything in one place.

For rehabilitants with acquired brain injury, it is important to have low-stimulus areas in a rehabilitation ward. Individual bedrooms provide a low-stimulus area, exercise options, privacy, and coping opportunities. As a rehabilitant with a lower limb amputation said,

Just single rooms, so you can also deal with your emotions.

##### Theme 2.2: Staff Aspects

Professionals working in a rehabilitation ward must have an empathic way of working, be able to support rehabilitants emotionally, and stimulate rehabilitants to practice. As an informal caregiver said about the desired attitude of professionals,

Striving for independence, in an active way.

Professionals must have good communication skills to communicate with rehabilitants and informal caregivers and within the team. Participants prefer small teams. This stimulates collaboration among professionals who feel they are responsible for the rehabilitation process.

##### Theme 2.3: Organizational Aspects

Participants see a high workload for the professionals and feel that, because of cutbacks in funding, they are mainly working towards a short length of stay. They are afraid of negative consequences for their rehabilitation, although they are in favor of efficiently organized rehabilitation to achieve optimal results.

##### Theme 2.4: Return Home

Although home visits during the rehabilitation can be confronting, participants find them useful. As an informal caregiver related to home visits,

Because then he could anticipate what it was of course. But it [home visit] was very painful for him, he found it terribly painful.

All participants are aware that rehabilitation continues after discharge. Therefore, a good and timely transfer between settings is necessary. Participants are not sure whether rehabilitation at home or on a ward is better at the start of the rehabilitation process. Some participants think the length of stay is too short. One informal caregiver said about a longer stay,

Well, I thought it would have been better. That he would feel safer. Yes, that's what I think. That this is just too short.

And others want to keep their time on the ward as short as possible, like a rehabilitant with multiple fractures,

Yes, the time I am here … I want to keep it as short as possible. Yes, I do.

## Discussion

In this article, for the first time, the perspectives of rehabilitants and informal caregivers regarding a Challenging Rehabilitation Environment are described. The themes identified can be divided into factors concerning the rehabilitation process and factors concerning organizational processes. Regarding the rehabilitation process, we found 9 themes: (1) rehabilitant, (2) rehabilitant centered, (3) informal caregivers, (4) communication, (5) exercise, (6) peer support, (7) daily schedule, (8) nutrition, and (9) eHealth. Four themes were identified regarding organizational processes: (1) environmental aspects, (2) staff aspects, (3) organizational aspects, and (4) return home.

Rehabilitants and informal caregivers experience a lack of attention for neuropsychiatric symptoms during the rehabilitation process, for example, altered stimulus processing, overburden, a decline of executive functions, dealing with emotions, loss of memory, and loss of initiative. They want professionals to provide information about these symptoms repeatedly and in different ways, for example, written, oral, or audio-visual. Recent literature has shown that neuropsychiatric symptoms like depression, disinhibition, and anxiety are highly prevalent in rehabilitants, and these symptoms are negatively associated with quality of life and home discharge after rehabilitation (16–20). Information about neuropsychiatric symptoms and their treatment may result in better rehabilitation outcomes. Therefore, we recommend that professionals in geriatric rehabilitation receive training on neuropsychiatric symptoms, which enables them to provide relevant information to rehabilitants and informal caregivers.

Participants in this study emphasized tailoring the whole rehabilitation process to their specific situation. They see value in being involved in the process of goal setting. Wade (21) and Holstege et al (22)describe that rehabilitation has to be tailored to the needs, goals, and wishes of the individual rehabilitant. In a recent meta-analysis, the importance of shared decision making was seen as a way to respect the preferences, values, and autonomy of rehabilitants (23). In the review of Vaalburg et al (24), the authors described the importance of involving rehabilitants in the process of setting meaningful goals. Tailoring the rehabilitation process to the personal situation of the rehabilitant, by means of goal setting is recommended, for example, when developing the treatment plan in the first week of admission.

Not all rehabilitants are aware that therapy includes all activities that promote rehabilitation rather than only activities with the physical therapist. Moreover, group training is a way to increase the intensity of therapy, and rehabilitants with group training experience are positive about this kind of therapy, whereas rehabilitants without experience cannot imagine it being effective. These factors seem to indicate the currently insufficient communication on these aspects and the types of therapy, although rehabilitants and informal caregivers emphasize the importance of good communication during the rehabilitation process. This includes communication about their rehabilitation process, diagnoses, and practical aspects such as the daily routine on the ward. Literature has shown that a lack of communication has a negative effect on the rehabilitation process, as the personal needs of rehabilitants are unclear to the professionals (25). Good communication is complex and there can be multiple barriers and facilitators related to organization, staff, and rehabilitant factors (26). Barriers can, for example, relate to mixed wards, the power imbalance between staff and rehabilitants, staff perception of time pressures, personality, lack of knowledge and skills regarding communication, or a rehabilitant's functional and medical status. Examples of facilitators are shared rooms, staff knowledge and utilization of communication strategies, and the personality of staff (26) This emphasizes the complexity and importance of communication, so rehabilitants know what to expect in a CRE and what they can do themselves. Therefore, when implementing a CRE, attention must be paid to all these aspects of communication. Consideration should be given to optimizing the physical environment and training staff in communication skills.

Participants in the current study see peer support as important for learning from each other, but also for support and recognition. For example, group training stimulates peer support and learning from each other, and peer support facilitates rehabilitants’ and caregivers’ adaptation to long-term disabilities (27,28). Group training and peer support during the rehabilitation process may result in better rehabilitation outcomes. We, therefore, recommend professionals stimulate group training, and peer support in a CRE.

Participants support the planning of therapy moments, so they can arrange visits around the therapy moments. Working with individualized timetables for rehabilitants and structured activities can increase a rehabilitants’ activity during inpatient rehabilitation (29). It is important to tailor the therapy moments to the needs of the rehabilitant, and therefore to follow the daily rhythm of rehabilitants by practicing meaningful tasks at the moment these tasks occur. We recommend providing clarity to rehabilitants about the day structure and possibilities to arrange visits. In addition, therapy moments should follow the rhythm of the rehabilitant during the day.

This is the first study into the perspectives of rehabilitants and informal caregivers regarding CRE. The strength of this study is the variety of informal caregivers and rehabilitants included. All participants had a recent experience with rehabilitation (and different diagnoses and ages), but were in different phases of their rehabilitation process, resulting in a good mix of participants. The use of focus groups stimulated the exchange of ideas, which also resulted in new ideas. Participants were asked for topics they thought were important for a CRE, even topics that were not mentioned by the researchers. In this way, it was ensured that all relevant topics were discussed and the internal validity of the study was increased. In this study, 15 rehabilitants and 6 informal caregivers from the Netherlands participated. In order to increase the external validity of the study, participants were included until data saturation was achieved. Although all rehabilitants rehabilitated in Dutch rehabilitation institutions, it is likely that results can be generalized to other countries.

A limitation of our study is that, due to COVID-19, we were unable to include more informal caregivers in focus groups and had to switch to telephone interviews. This limited the discussion between participants, but these informal caregivers were able to express their own ideas regarding CRE without interruption. The results of these telephone interviews were in line with the results of the focus groups. Therefore, we expect that this adjustment had no effect on the results of this study and that the outcomes are broadly supported by informal caregivers and rehabilitants.

The second limitation of our study is that most of the informal caregivers were caregivers of a rehabilitant with a brain injury. The perspectives of the informal caregiver from a rehabilitant after trauma were in line with the perspectives of the informal caregivers from rehabilitants with brain injury. Also, the perspectives of the rehabilitants themselves with and without brain injury were comparable. Therefore, we expect that this distribution had no effect on the results of this study.

## Conclusion

Based on this study, 13 themes were identified for modeling CRE from the perspectives of rehabilitants and informal caregivers. It is important to stimulate rehabilitants to be as active as possible during their rehabilitation and to tailor the rehabilitation process to the individual rehabilitant. Therefore, organizing excellent rehabilitation care requires a thorough understanding of the concept of CRE, since it is a complex and comprehensive concept that concerns the whole rehabilitation process. Its effectiveness and efficiency should be researched in prospective studies.

## Supplemental Material

sj-docx-1-jpx-10.1177_23743735231151532 - Supplemental material for A Qualitative Study Exploring Rehabilitant and Informal Caregiver Perspectives of a Challenging Rehabilitation Environment for Geriatric Rehabilitation Click here for additional data file. Supplemental material, sj-docx-1-jpx-10.1177_23743735231151532 for A Qualitative Study Exploring Rehabilitant and Informal Caregiver Perspectives of a Challenging Rehabilitation Environment for Geriatric Rehabilitation by Lian M J Tijsen, Els W C Derksen, Wilco P Achterberg and Bianca I Buijck in Journal of Patient Experience

## Appendix 1. Topic list

- What do you think about how your rehabilitation process was structured?
- What made you feel that you were or were not stimulated to work on your rehabilitation yourself?
- What do you think are important components to make your rehabilitation a success?
- Additional topics to discuss in relation to CRE:
  - Therapy intensity
  - Task-oriented exercise
  - Group training
  - Patient-regulated exercise
  - Learning styles and approach
  - Goal setting
  - Team dynamics (multidisciplinary, interdisciplinary)
  - Technologies
  - Enriched environment
  - Informal caregiver participation
  - Diagnoses
  - Nutrition
